# A new spider species, *Heser stoevi* sp. nov., from Turkmenistan (Araneae: Gnaphosidae)

**DOI:** 10.3897/BDJ.4.e10095

**Published:** 2016-08-31

**Authors:** Christo Deltshev

**Affiliations:** ‡ National Museum of Natural History, Bulgarian Academy of Sciences, 1 Tsar Osvoboditel Blvd., Sofia, Bulgaria

**Keywords:** taxonomy, description, Koytendag Mountains, genital characters

## Abstract

**Background:**

The genus *Heser* (Araneae, Gnaphosidae) belongs to the *Zelotes* group, and is currently known to comprise 10 species distributed in Africa, Asia, Europe and North America. The type species is *Heser malefactor* Tuneva, 2004 from Kazakhstan.

**New information:**

A new spider species, *Heser stoevi* sp. nov. (male and female) found in Koytendag Mountains, southeastern Turkmenistan is described and illustrated. It is morphologically close to *H. aradensis* (Levy, 1998) from Israel but can be readily distinguished by the longer embolus, the smaller and flattened conductor, the lack of retrolateral tibial apophysis, all this coupled with very distinctive female copulatory organs. The new species was found under stones in arid grassland habitats.

## Introduction

The genus *Heser* (Araneae, Gnaphosidae) belongs to the *Zelotes* group, which comprises genera of small to medium-sized spiders (typical size ranges from 2 to 10 mm) with preening distal combs on metatarsi III and IV (Murphy 2008). This character is not possessed by any other gnaphosid group. The genus is delimited by Tuneva (2004) with the description of the type species *Heser malefactor* Tuneva, 2004 from Kazakhstan. In this work, she proposed also two new combinations, both *ex. Zelotes - H. aradensis* (Levy, 1998) from Israel, and *H. infumatus* (O. Pickard-Cambridge, 1872) from Egypt, Israel and Tanzania. Later, Bosselaers (2010) and Senglet (2012), described two other species, respectively *H. vijayanagara* Bosselar, 2010 from India and *H. hispanus* Senglet, 2012 from Spain. The latter author proposed also the new combinations (all *ex. Zelotes*): *H. bernardi* (Marinaro, 1967) from Spain, Algeria, *H. bonneti* (Marinaro, 1967) from Algeria, *H. hierosolymitanus* (Levy, 1998) from Israel, *H. nilicola* (O. Pickard-Cambridge, 1874) from the Mediterranean region, Canary Is, Burkina Faso, USA and Mexico, and *H. schmitzi* (Kulczyński, 1899) from Spain, Madeira, Canary Is. and USA (World Spider Catalog 2016).

Thus, the genus is currently known to comprise 10 species ranging from India to the USA. Here, we describe a new species of *Heser* collected from two closely situated localities in the Koytendag State Nature Reserve, Koytentag Mts, southeastern Turkmenistan. The material has been collected in the course of a Rapid Environmental Assessment survey carried out by an international team of zoologists in Koytendag Mts. (= Köýtendag, Kugitang, Koitendag, Kugitangtau, Kugitang-Tay, Kugitangtou).

## Materials and methods

The specimens were hand-collected under stones. Coloration is described from alcohol-preserved specimens. Male palps and female genitalia were examined and illustrated after dissection from spiders’ bodies. All photos were taken with the aid of Panasonic DMC-FS62 digital camera mounted on Wild M5A stereomicroscope. Measurements of the legs are taken from the dorsal side. Total length of the body includes the chelicerae. All measurements are in mm. Abbreviations: AME – anterior median eyes; ALE – anterior lateral eyes; E – embolus; C – conductor; MA – median apophysis; PLE – posterior lateral eyes; PME – posterior median eyes; SD – sperm duct; ST – spermatheca. The type specimens are deposited in the National Museum of Natural History (NMNHS), Sofia, Bulgaria.

Material used for comparison: *Heser aradensis* (Levy, 1998), 1 male, Israel, nr. Nehusha, 14.05.2002, leg Y. Mandelik, det G. Levy; 1 male, Golan, Ramat Magshimim, 06.1998, leg I. Warburg, det G. Levy; 1 female, Golan, Geshur, 06.1998, leg I. Warburg, det G. Levy, Collection of Hebrew University of Jerusalem, Zoological Museum.

## Taxon treatments

### Heser stoevi

Deltshev sp. n.

urn:lsid:zoobank.org:act:E4D7D5A0-D649-4F5E-9360-D0488D73EEE8

#### Materials

- **Type status:** Holotype. **Occurrence:** recordedBy: P. Stoev; individualCount: 1; sex: male; lifeStage: adult; **Taxon:** scientificName: Heser stoevi; family: Gnaphosidae; taxonRank: species; scientificNameAuthorship: Deltshev; **Location:** continent: Asia; country: Turkmenistan; stateProvince: Lebap; county: Koytendag District; locality: v. Hojeypil, around the Dinosaurs tracks Site; verbatimElevation: 1150; decimalLatitude: 37 56.443; decimalLongitude: 66 37.597; **Identification:** identifiedBy: Christo Deltshev; dateIdentified: 2015; **Event:** samplingProtocol: hand collecting; eventDate: 05/25/2015; habitat: under stones; **Record Level:** institutionID: NMNHS; collectionID: Archnida; basisOfRecord: PreservedSpecimen
- **Type status:** Paratype. **Occurrence:** recordedBy: P. Stoev; individualCount: 1; sex: female; lifeStage: adult; **Taxon:** scientificName: Heser stoevi; family: Gnaphosidae; taxonRank: species; scientificNameAuthorship: Deltshev; **Location:** continent: Asia; country: Turkmenistan; stateProvince: Lebap; county: Koytendag District; locality: v. Hojeypil, around the Dinosaurs tracks Site; verbatimElevation: 1150; decimalLatitude: 37 56.443; decimalLongitude: 66 37.597; **Identification:** identifiedBy: Christo Deltshev; dateIdentified: 2015; **Event:** samplingProtocol: hand collecting; eventDate: 05/25/2015; habitat: under stones; **Record Level:** institutionID: NMNHS; collectionID: Archnida; basisOfRecord: PreservedSpecimen
- **Type status:** Paratype. **Occurrence:** recordedBy: P. Stoev; individualCount: 1; sex: female; lifeStage: adult; **Taxon:** scientificName: Heser stoevi; family: Gnaphosidae; taxonRank: species; scientificNameAuthorship: Deltshev; **Location:** continent: Asia; country: Turkmenistan; stateProvince: Lebap; county: Koytendag District; locality: v. Garlyk, around cave Gulshirin (= Geofizicheskaya); verbatimElevation: 860; decimalLatitude: 37 40.394; decimalLongitude: 66 23.698; **Identification:** identifiedBy: Christo Deltshev; dateIdentified: 2015; **Event:** samplingProtocol: hand collecting; eventDate: 05/28/2015; habitat: under stones; **Record Level:** institutionID: NMNHS; collectionID: Archnida; basisOfRecord: PreservedSpecimen

#### Description

Male holotype: Total length 5.25; prosoma, length 2.55, width 1.80; sternum length 1.43, width 1.05; chelicerae, length 0.75, width 0.30; opisthosoma, length 5.25. Carapace uniformly brown, with a fovea in the posterior half (Fig. 1a, c). All eyes subequal, AME circular, separated from each other by their own diameter, ALE oval, touching AME. PME oval to subtriangular, touching, larger than AME. PLE oval, slightly smaller than ALE, separated from PME by more of one PLE diameter. Clypeus vertical, large one diameter of AME. Chelicerae brown, with a few scattered thin setae on anterior surface, promarginal rim with three very small teeth spaced closely to fang base, retromarginal rim spineless. Sternum smooth, yellow brown, shield-shaped with a thin border (Fig. 1b). Abdomen grey with frontal row of curved hairs and a scutum covering about 10% of abdominal dorsal surface area (Fig. 1a, c). Legs yellowish to yellow-brown (Fig. 1a, c), leg formula 4123, measurements as in Table 1.

Male palp (Fig. 2a, b, c, Fig. 5a, c, e): Distinctive retrolateral tibial apophysis is not presented, hence distal part of tibia retrolateral is strongly chitinized and dark colored. The embolus is slender, basally prolaterally inserted, extends across the broad and flat conductor (sensu Bosselaers 2010), circling more than half of the tegulum. Median apophysis large, hook-shaped.

Female paratype: Total length 6.00; prosoma, length 2.63, width 1.80; sternum length 1.50, width 1.05; opisthosoma, length 3.38 (Fig. 3a, c). All characters as described for male. Leg measurements as in Table 2​.

Epigyne and vulva (Figs 4a, b, 6a, b): characterized by well-developed copulatory openings, leading to coiled insemination ducts, connected with coiled spermathecae.

#### Diagnosis

The somatic characters of the new species correspond to those of the genus *Heser*, but the genitalia are distinctive and separate well the new species from all other congeners. Morphologically, the new species resembles *H. aradensis* (Fig. 1b, d) but the male has longer embolus, and smaller and flattened conductor (sensu Bosselaers 2010), as well as lacks distinctive retrolateral tibial apophysis (Fig. 2a, c, e). Female has distinctive epygine and vulva, characterized by well-defined copulatory openings leading to coiled insemination ducts, connected with coiled spermathecae (see Figs 4c, d, 6c, d for comparison).

#### Etymology

Named in honour of the Bulgarian zoologist Pavel Stoev, who collected the species; name in genitive case.

#### Distribution

**Figure 7.**
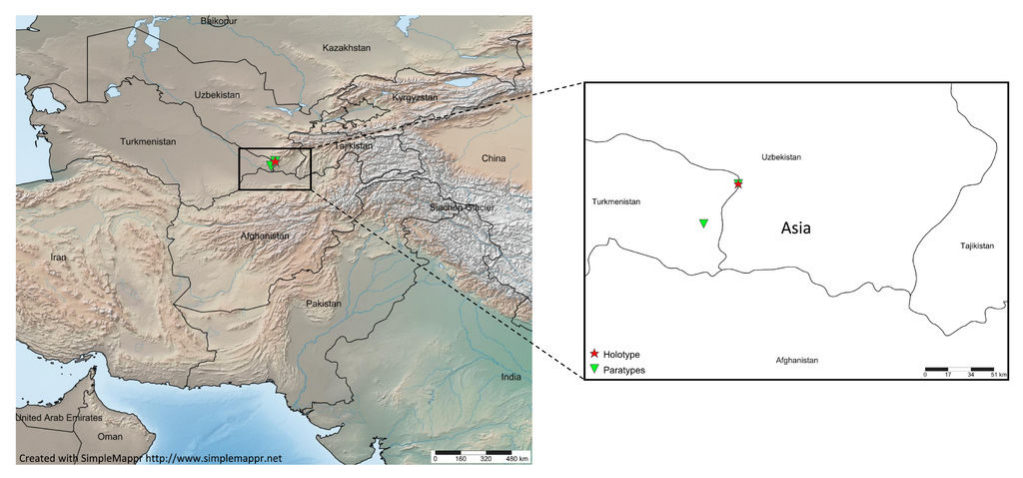
Distribution map of *Heser stoevi* sp. n.

The species is hitherto known only from two closely situated localities in the western slope of Koytentag Mts, southeastern Turkmenistan (Fig. 7).

## Supplementary Material

XML Treatment for Heser stoevi

## Figures and Tables

**Figure 1a.**
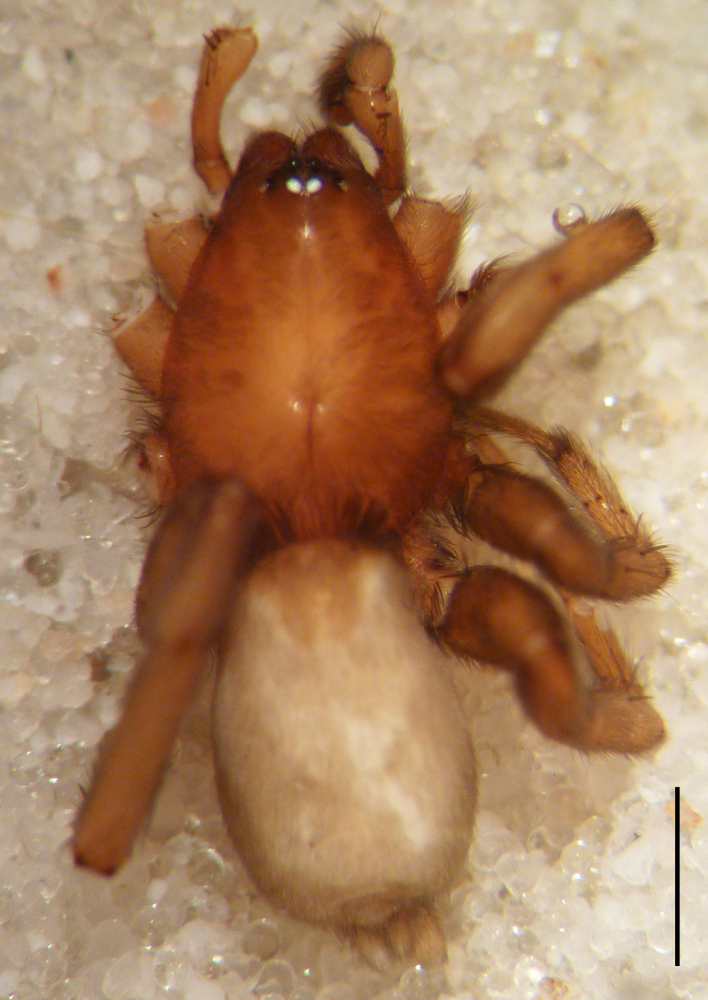
*Heser stoevi* sp. n., holotype, dorsal view.

**Figure 1b.**
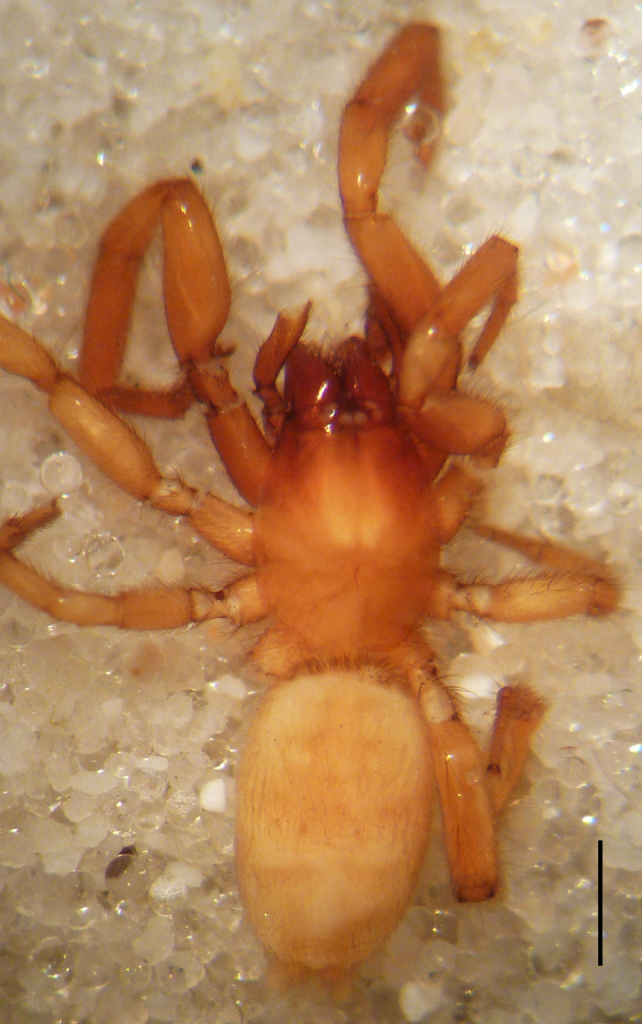
*Heser aradensis*, dorsal view.

**Figure 1c.**
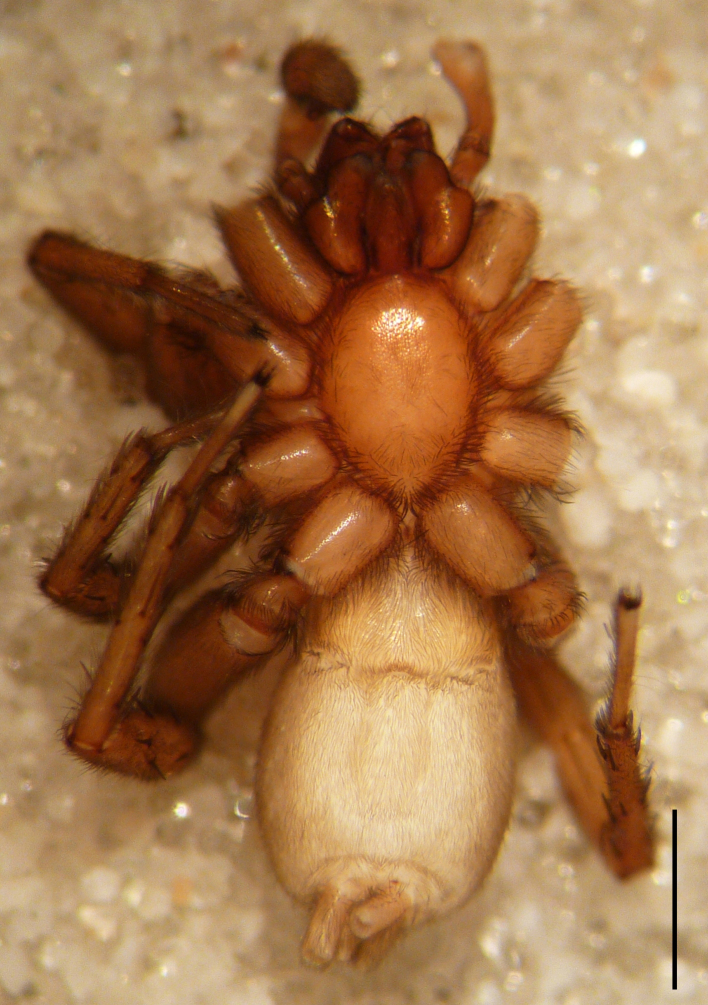
*Heser stoevi* sp. n., holotype, ventral view.

**Figure 1d.**
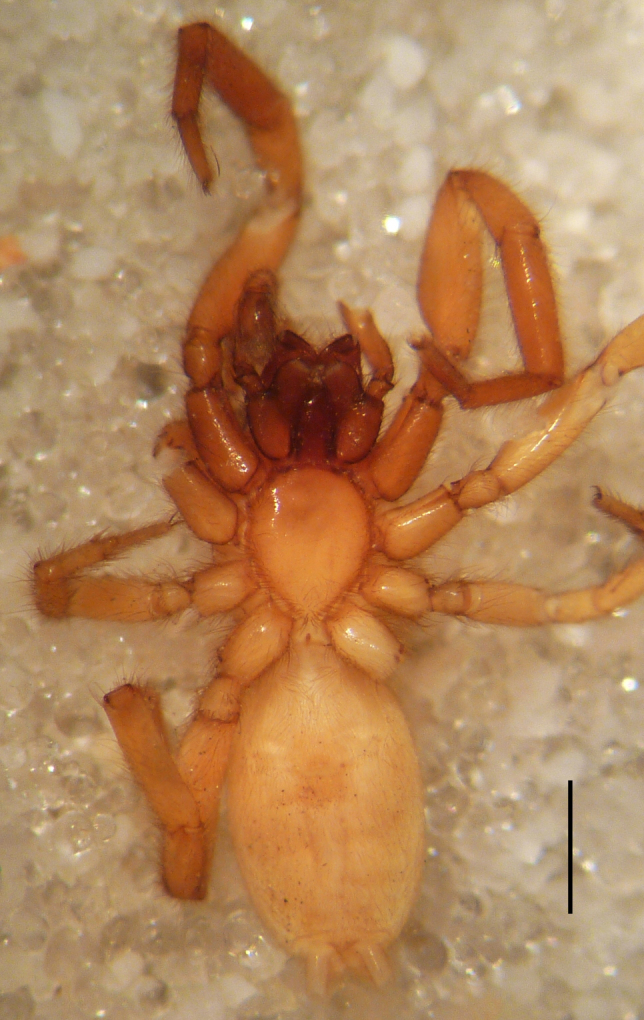
*Heser aradensis*, ventral view.

**Figure 2a.**
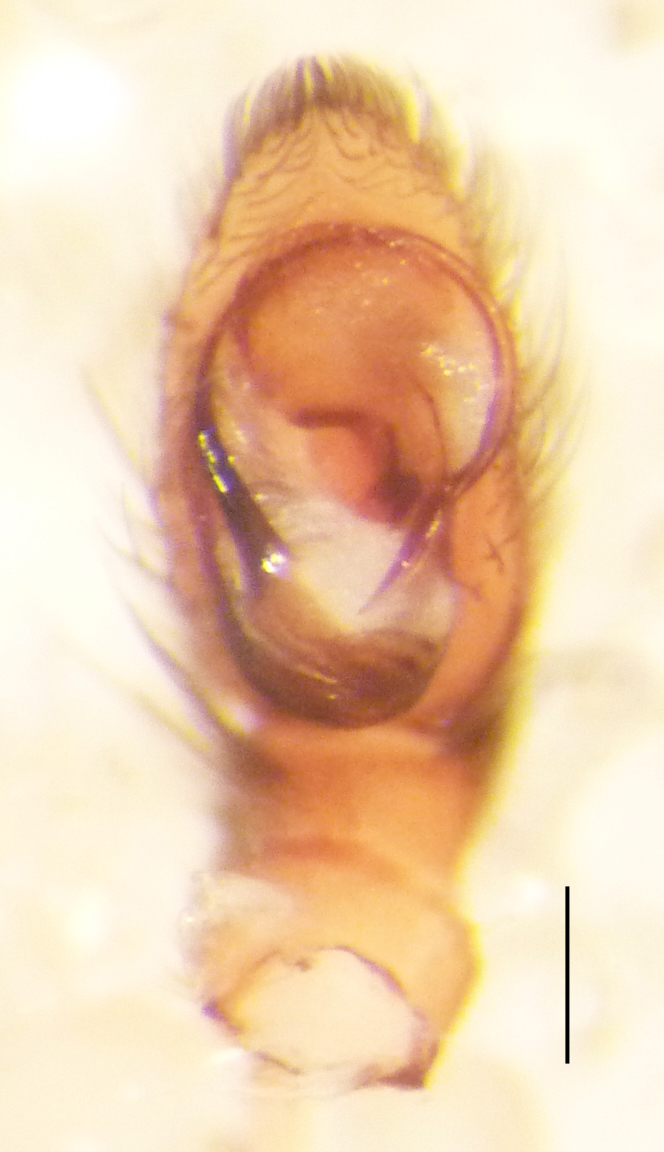
*Heser stoevi* sp. n., holotype, ventral view.

**Figure 2b.**
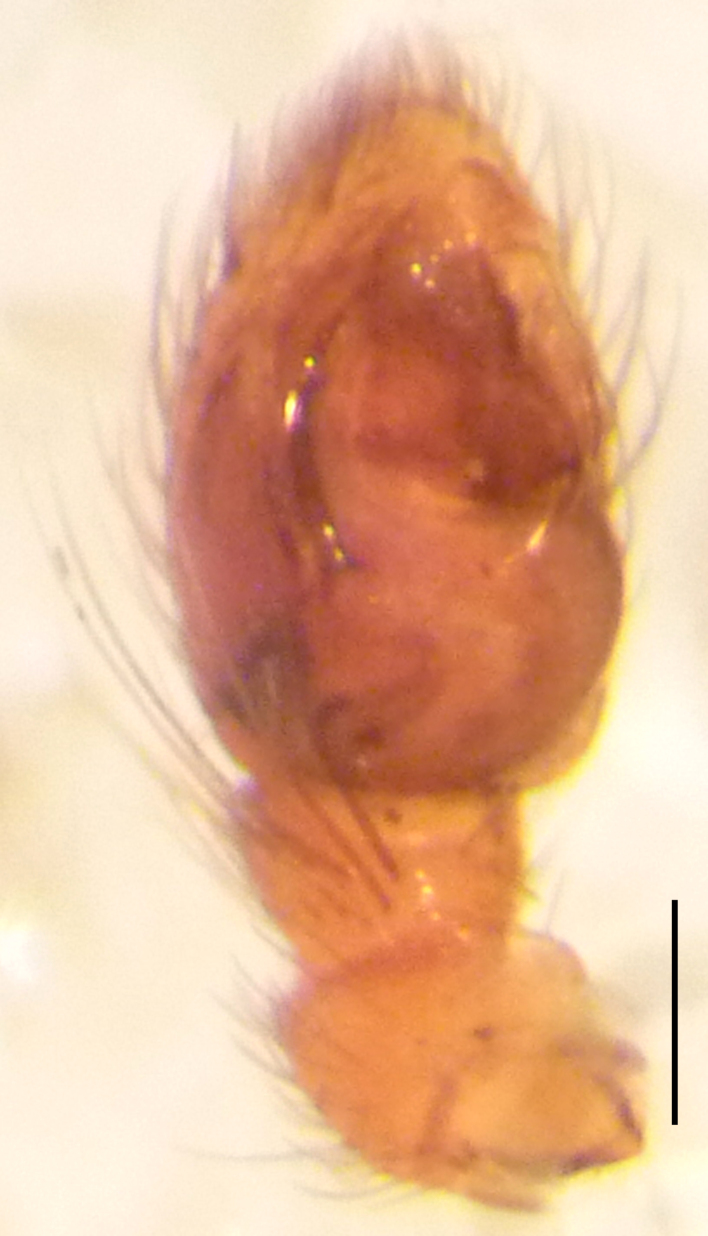
*Heser aradensis*, ventral view.

**Figure 2c.**
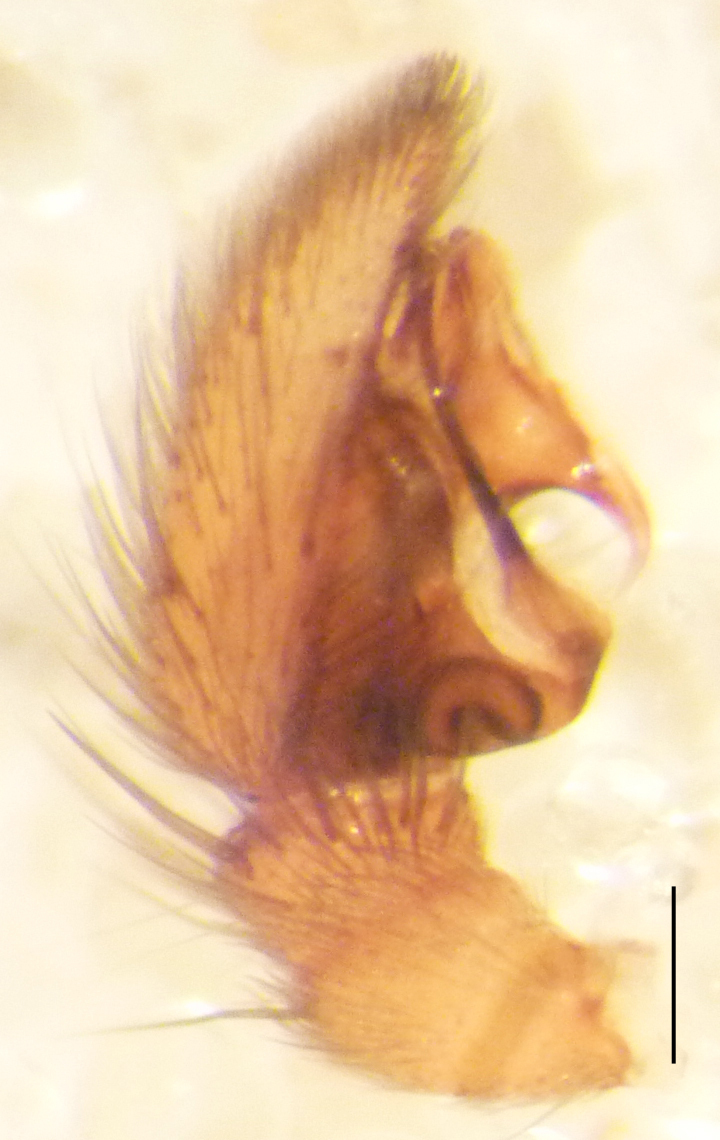
*Heser stoevi* sp. n., holotype, prolateral view.

**Figure 2d.**
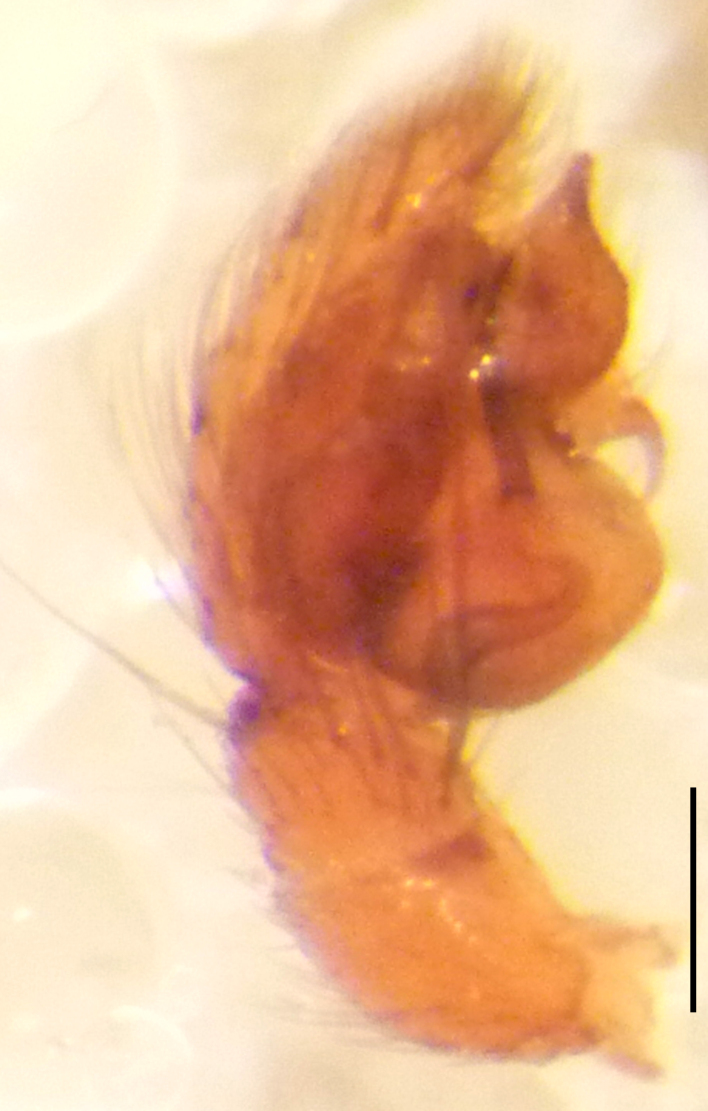
*Heser aradensis*, prolateral view.

**Figure 2e.**
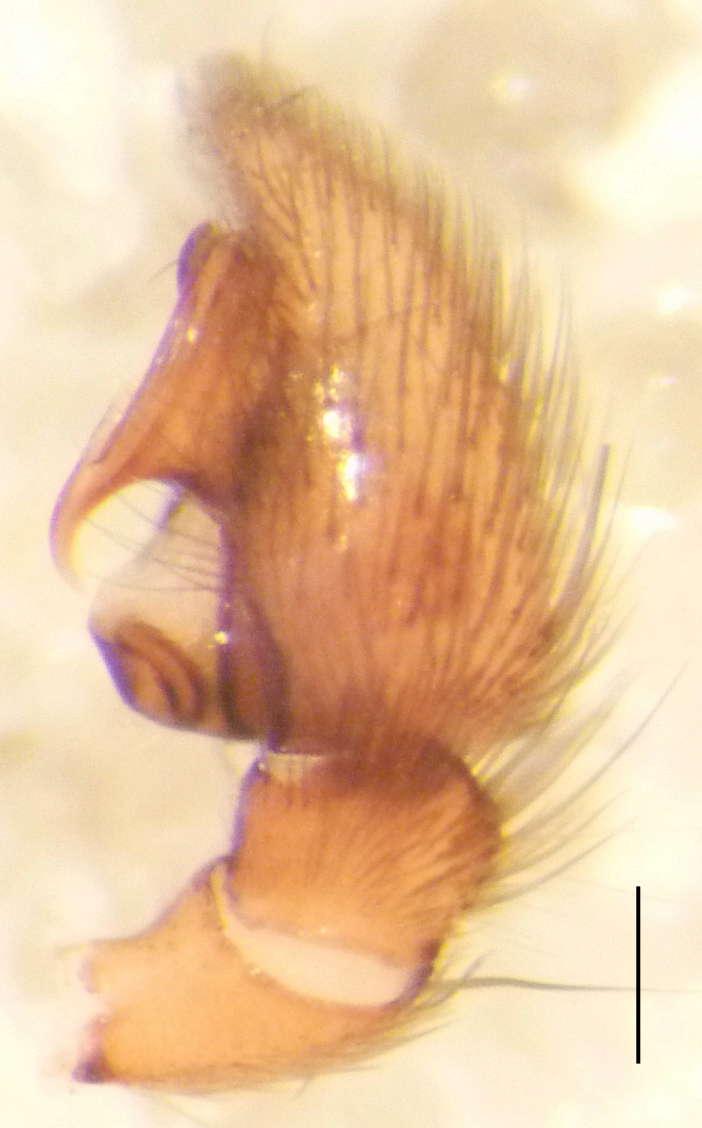
*Heser stoevi* sp. n., holotype, retrolateral view.

**Figure 2f.**
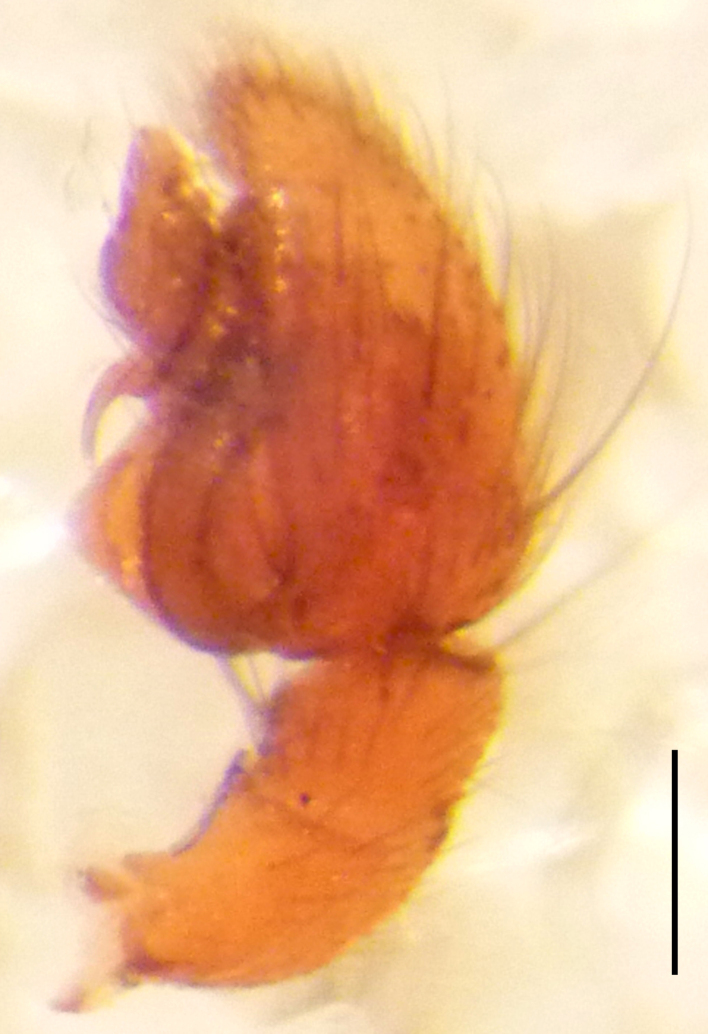
*Heser aradensis*, retrolateral view.

**Figure 3a.**
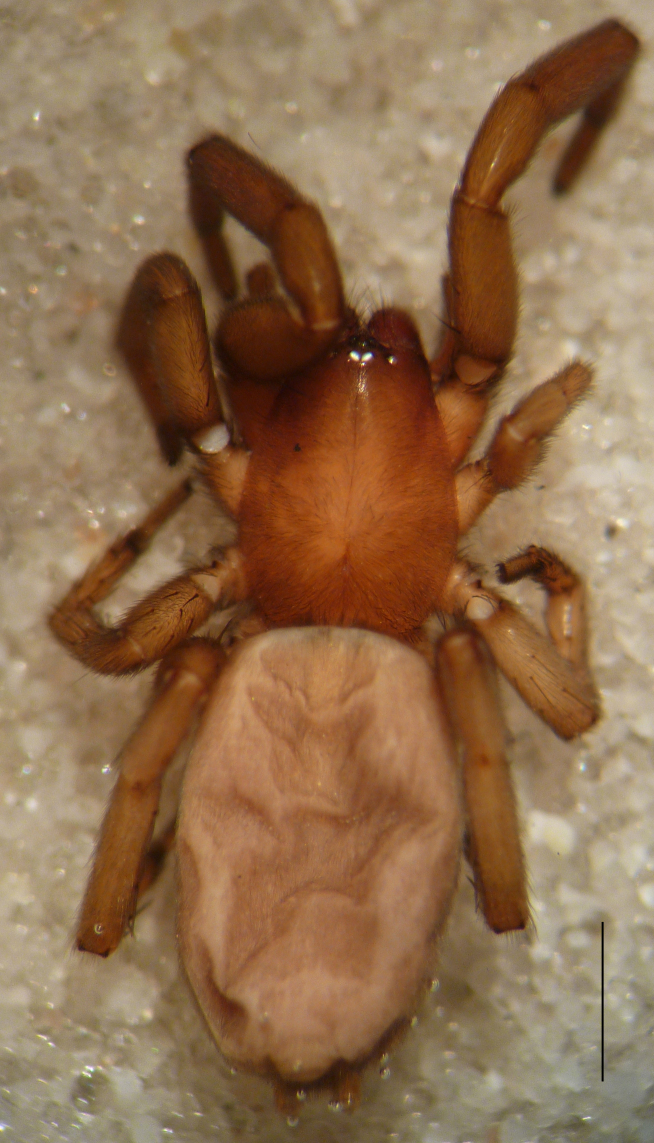
*Heser stoevi* sp. n., paratype, dorsal view.

**Figure 3b.**
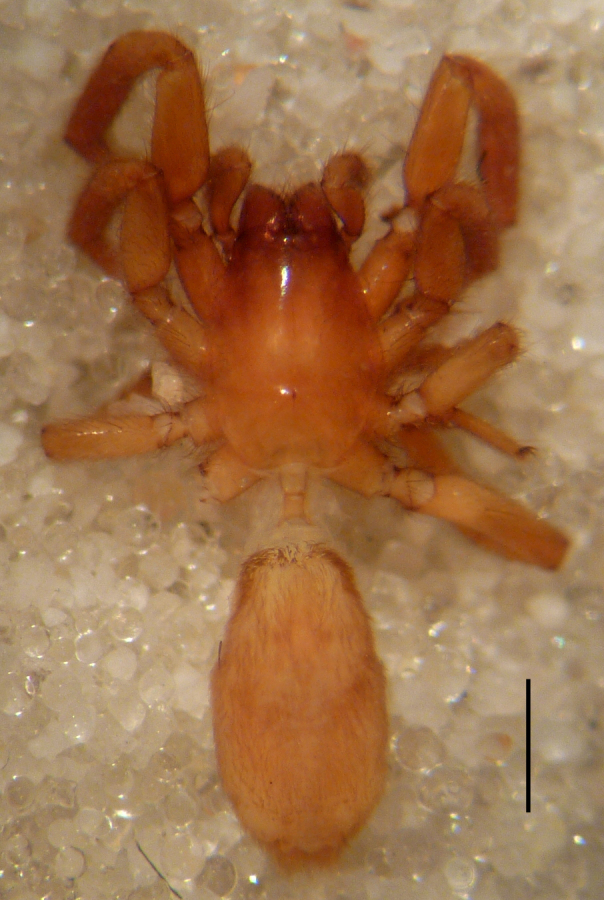
*Heser aradensis*, dorsal view.

**Figure 3c.**
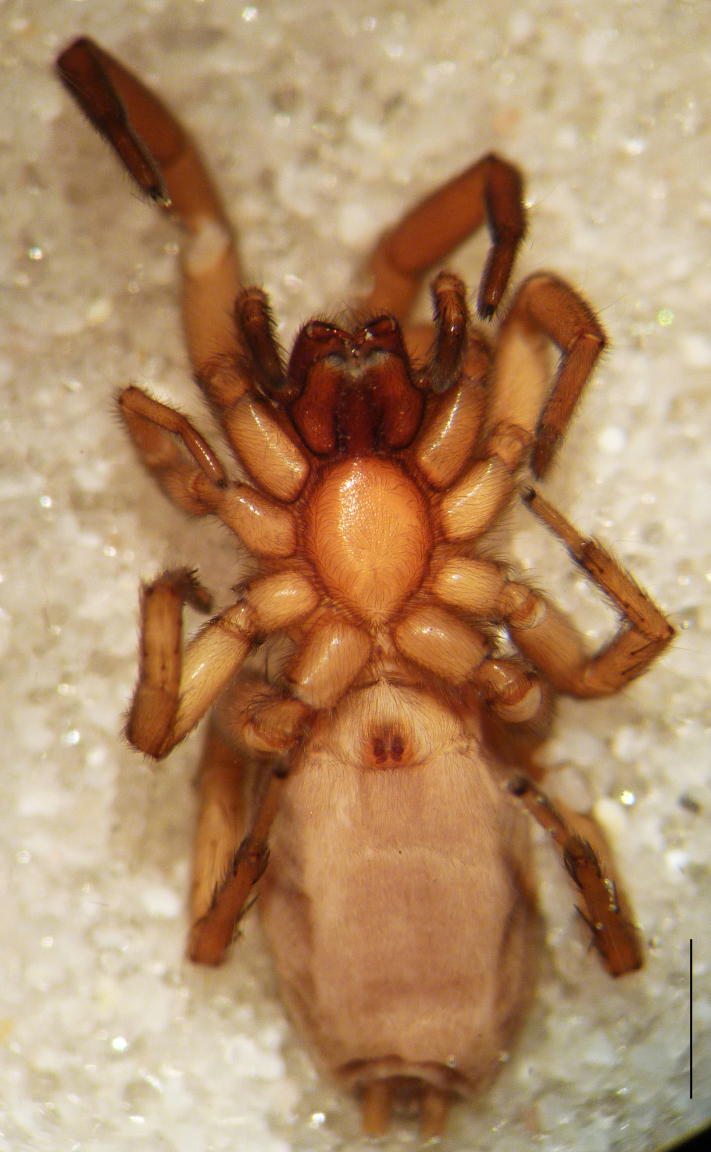
*Heser stoevi* sp. n., paratype, ventral view.

**Figure 3d.**
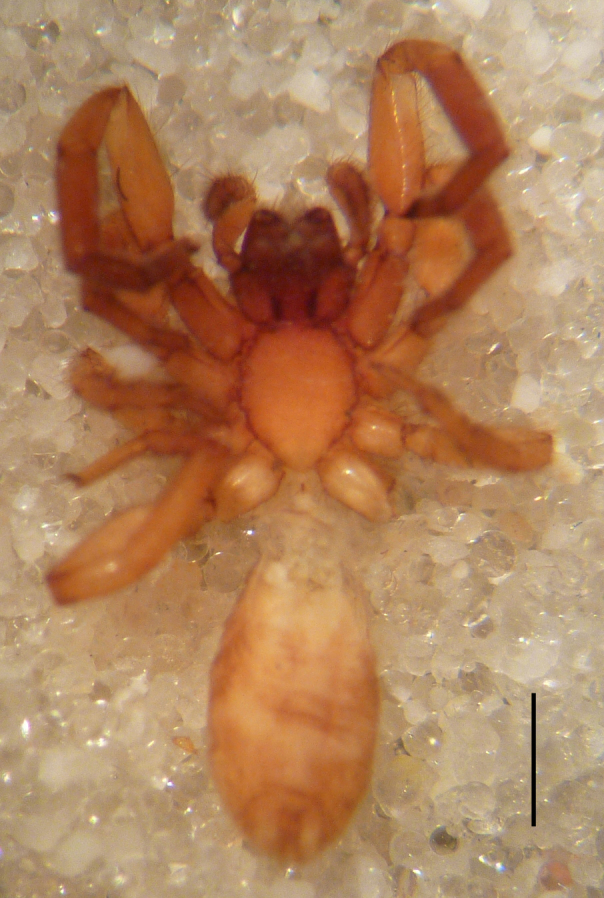
*Heser aradensis*, ventral view.

**Figure 4a.**
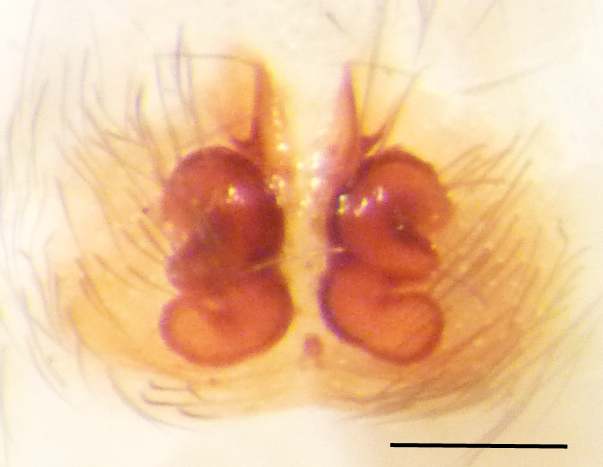
*Heser stoevi* sp. n., paratype, ventral view.

**Figure 4b.**
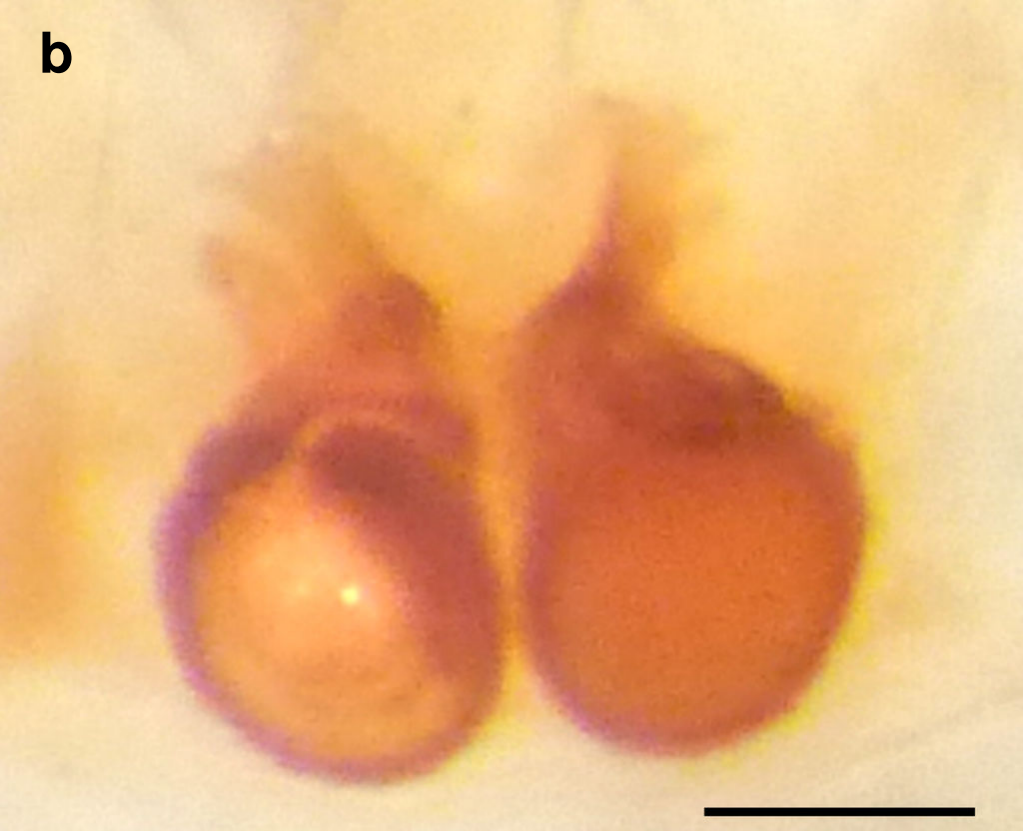
*Heser aradensis*, ventral view.

**Figure 4c.**
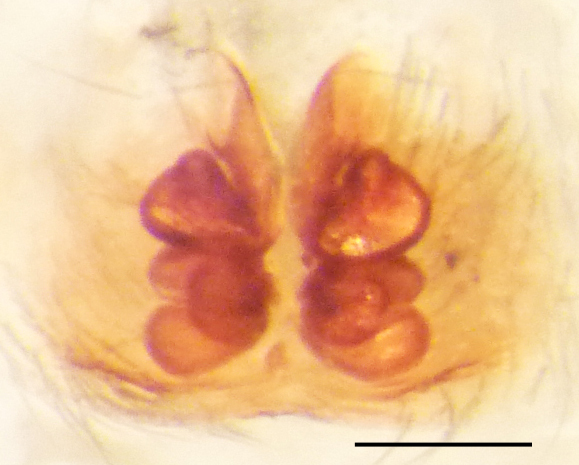
*Heser stoevi* sp. n., paratype, dorsal view.

**Figure 4d.**
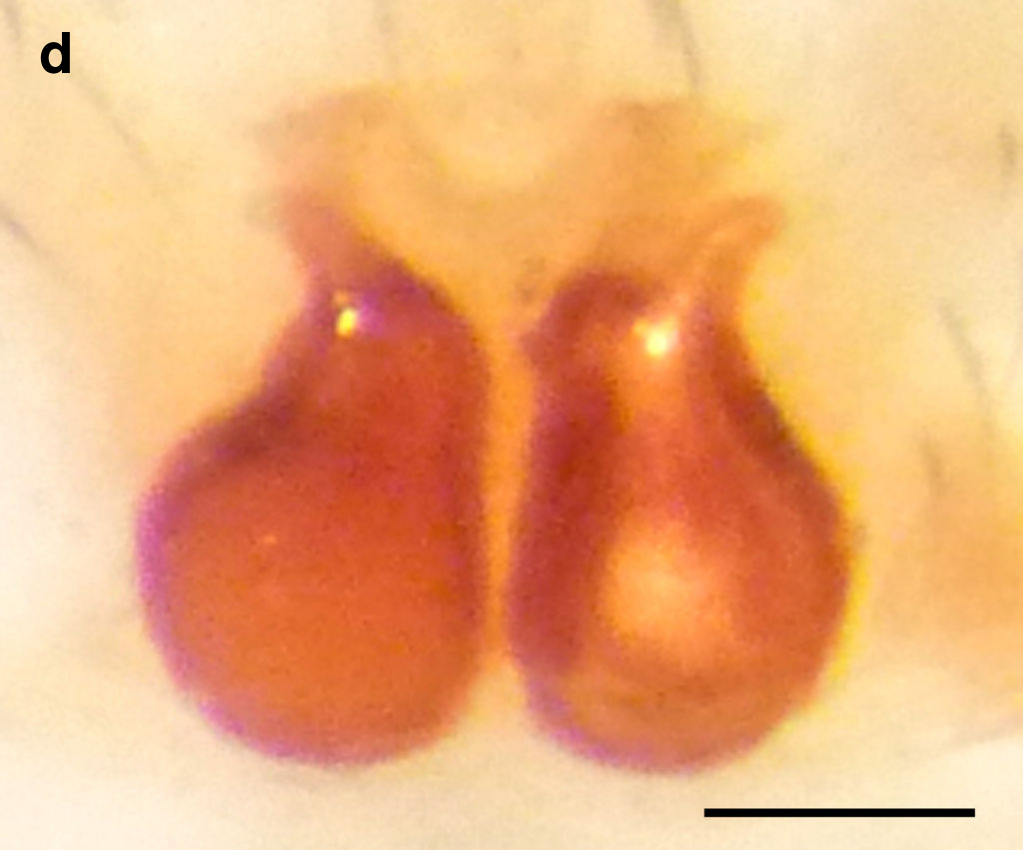
*Heser aradensis*, dorsal view.

**Figure 5a.**
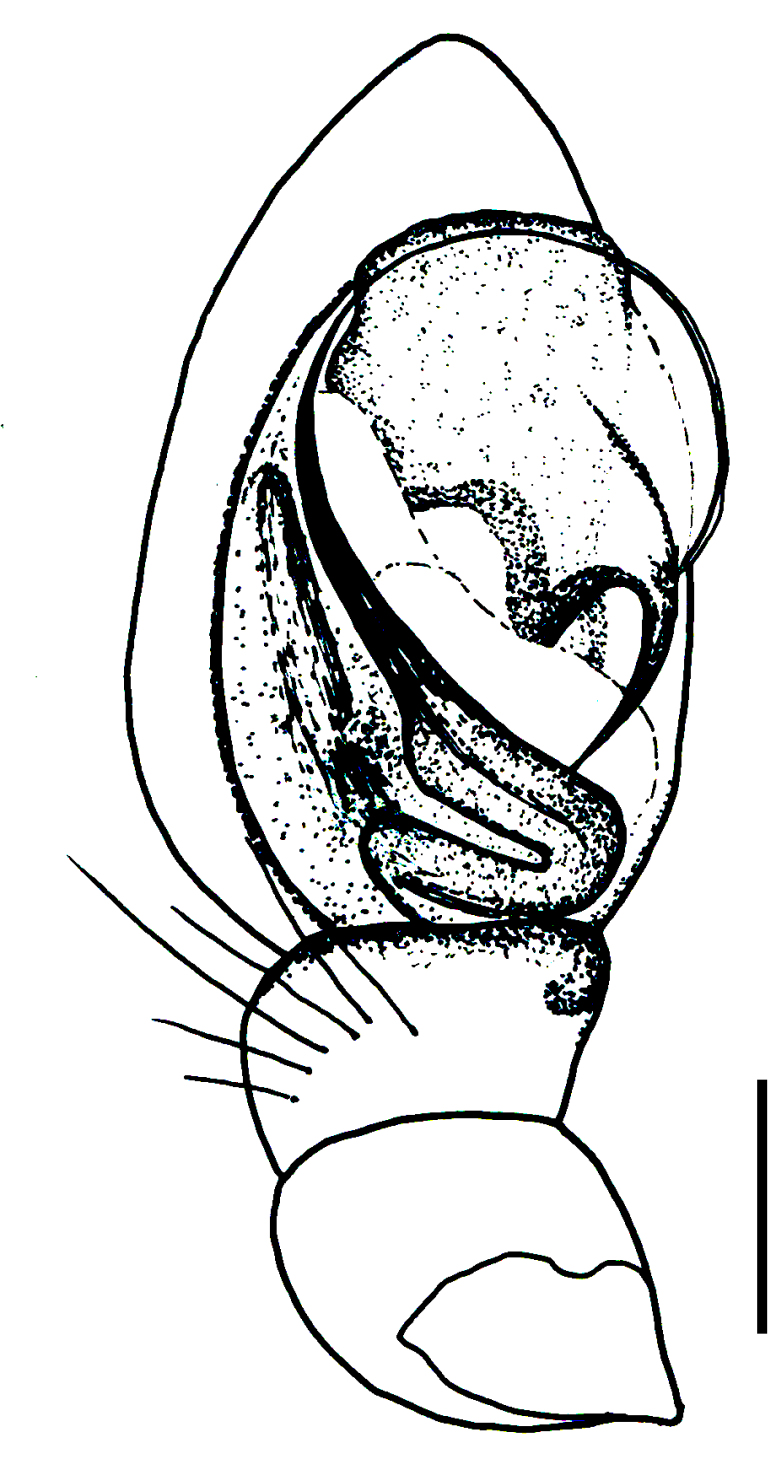
*Heser stoevi* sp. n., holotype, ventral view.

**Figure 5b.**
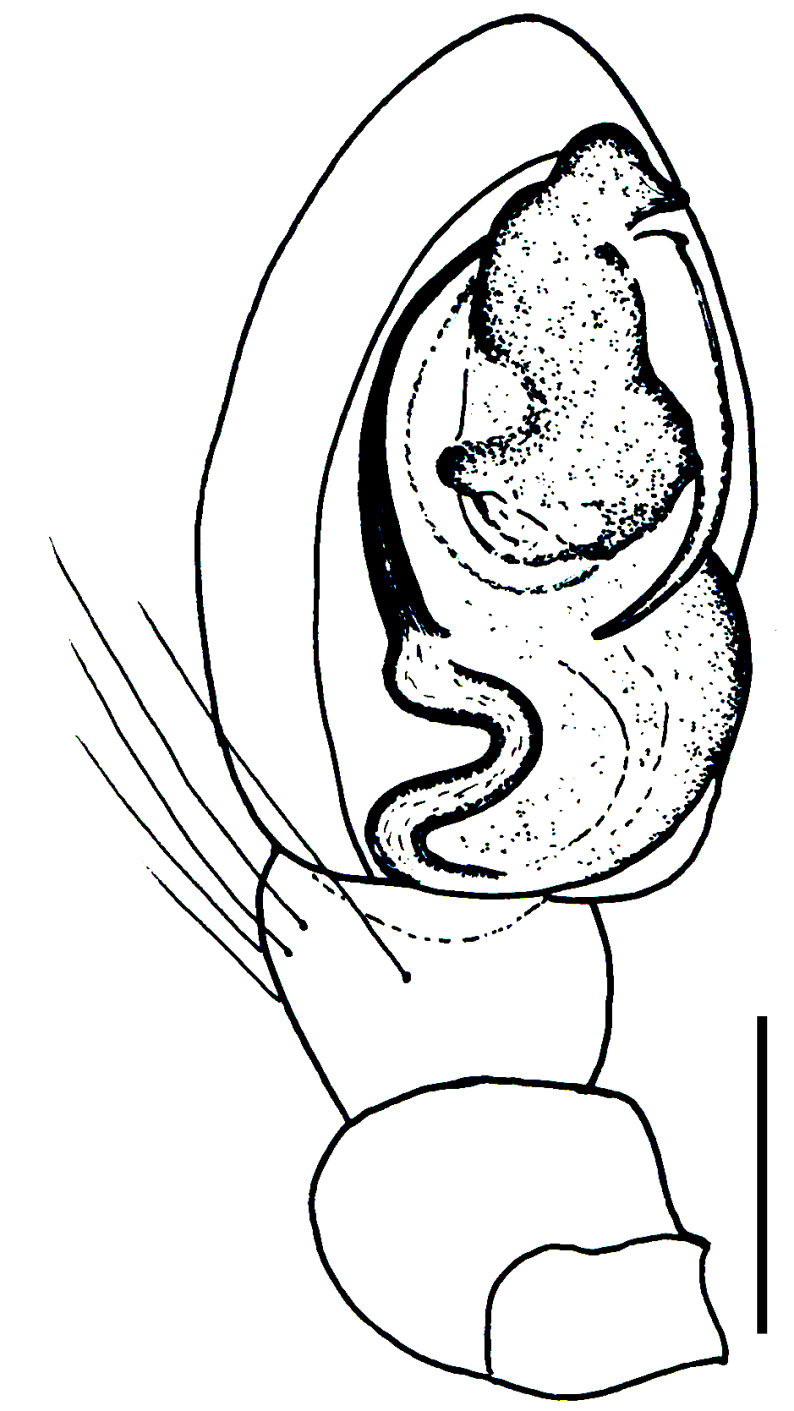
*Heser aradensis*, ventral view.

**Figure 5c.**
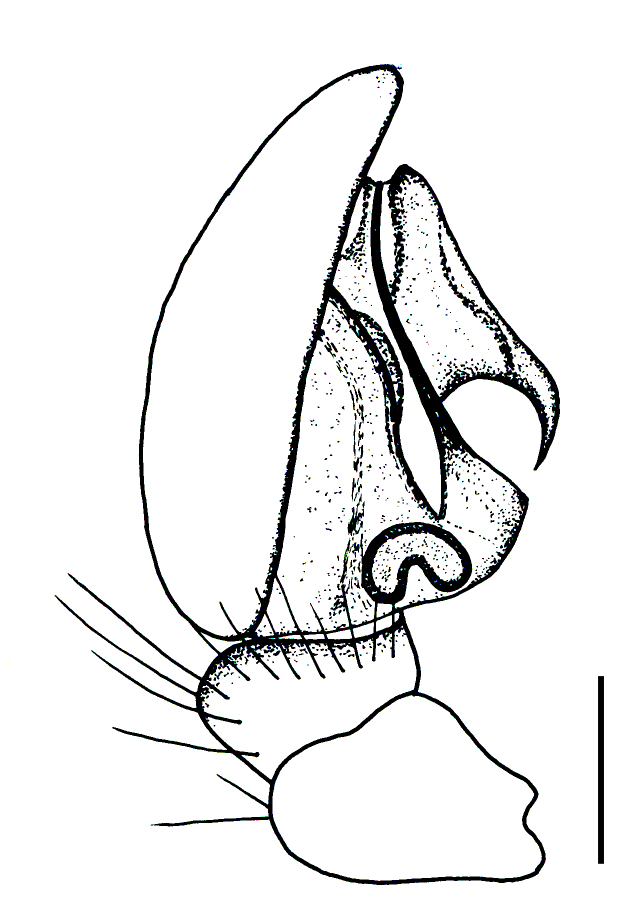
*Heser stoevi* sp. n., holotype, prolateral view.

**Figure 5d.**
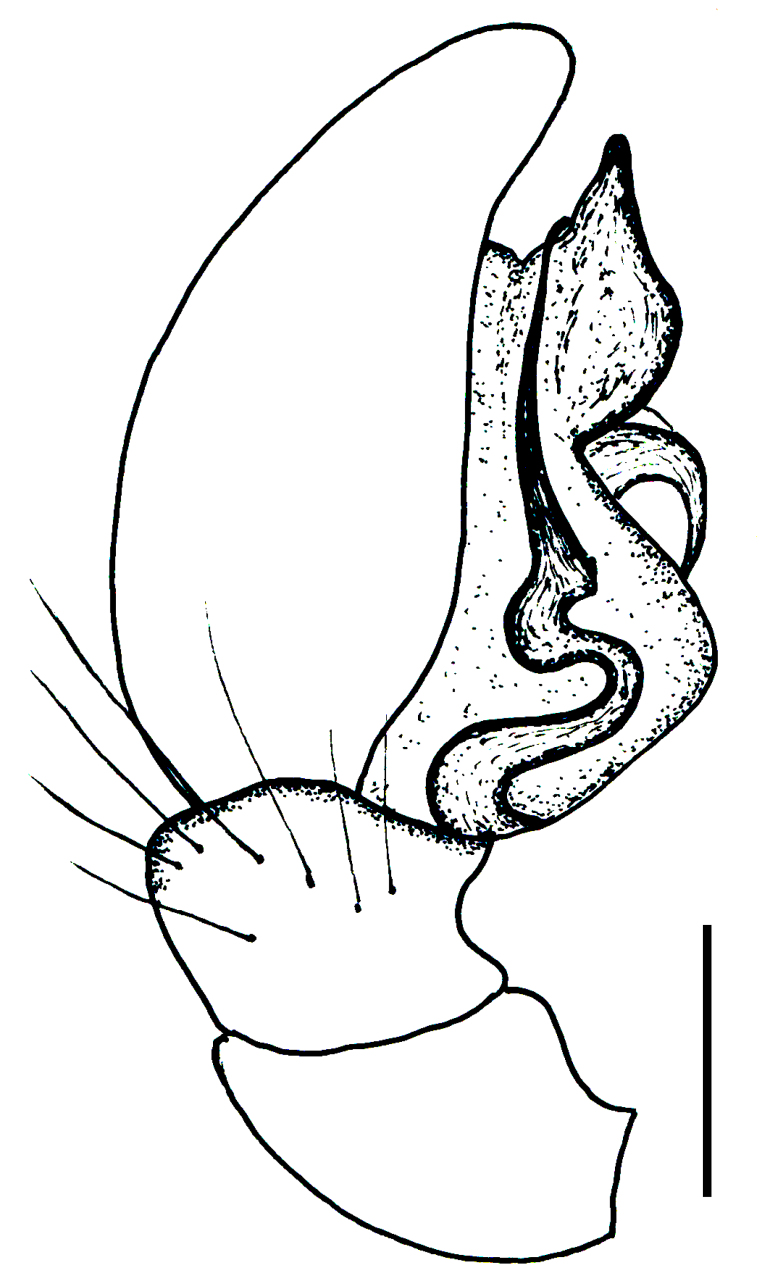
*Heser aradensis*, prolateral view.

**Figure 5e.**
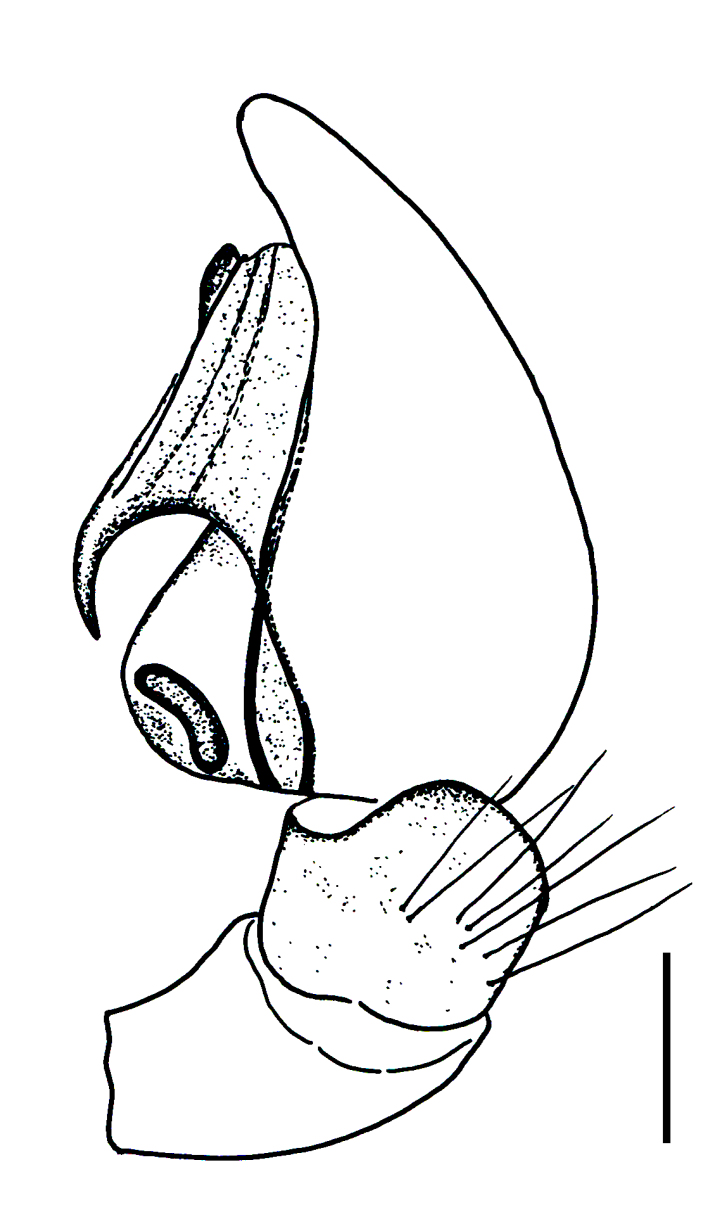
*Heser stoevi* sp. n., holotype, retrolateral view.

**Figure 5f.**
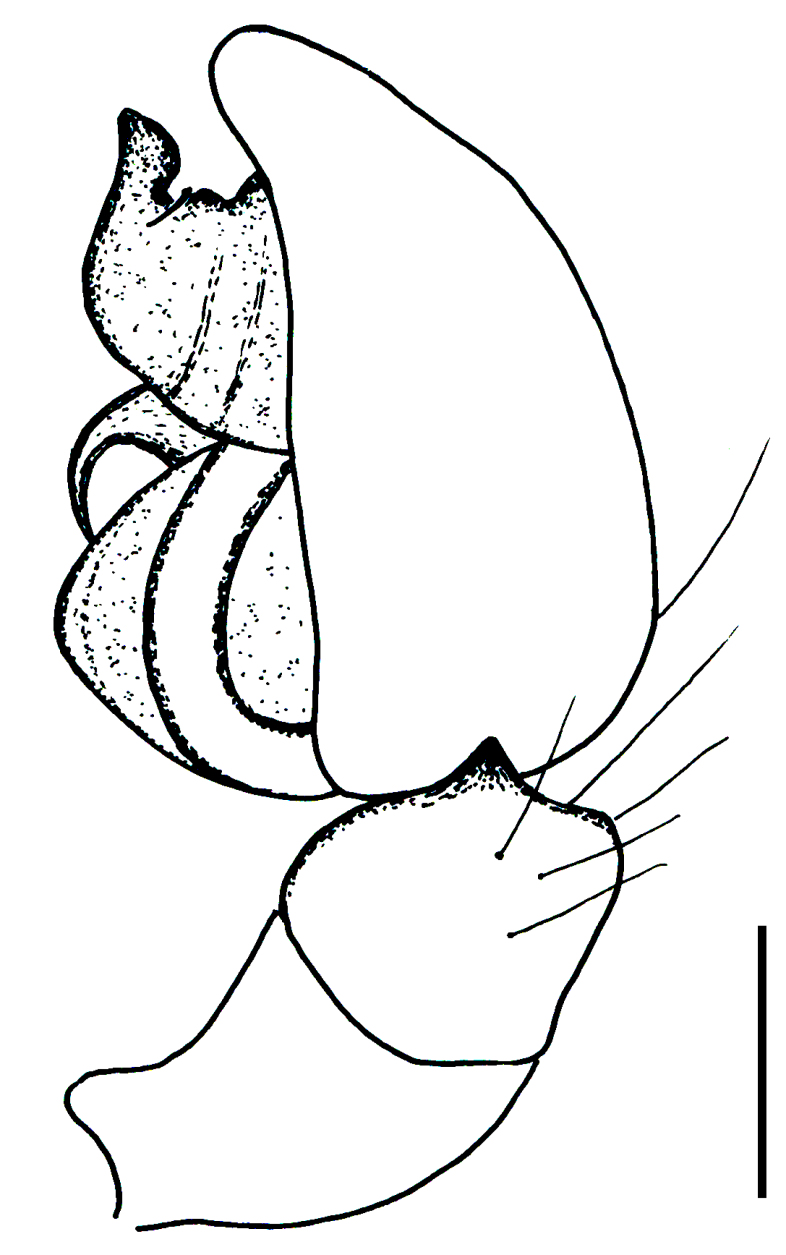
*Heser aradensis*, retrolateral view.

**Figure 6a.**
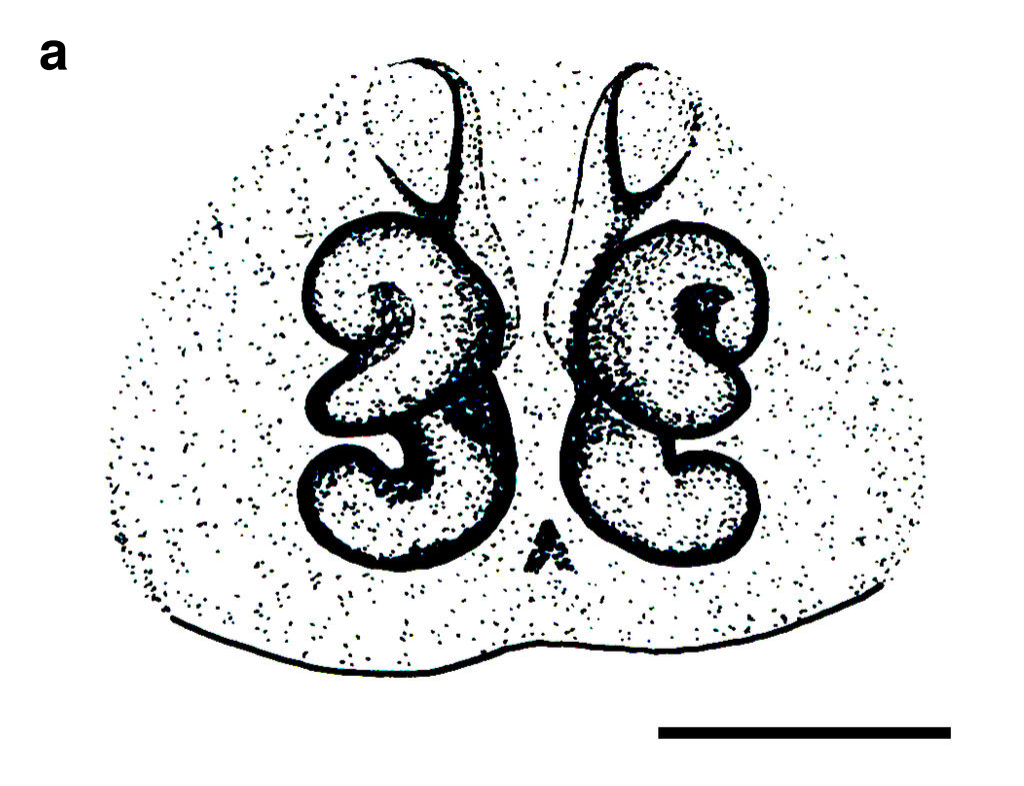
*Heser stoevi* sp. n., paratype, ventral view.

**Figure 6b.**
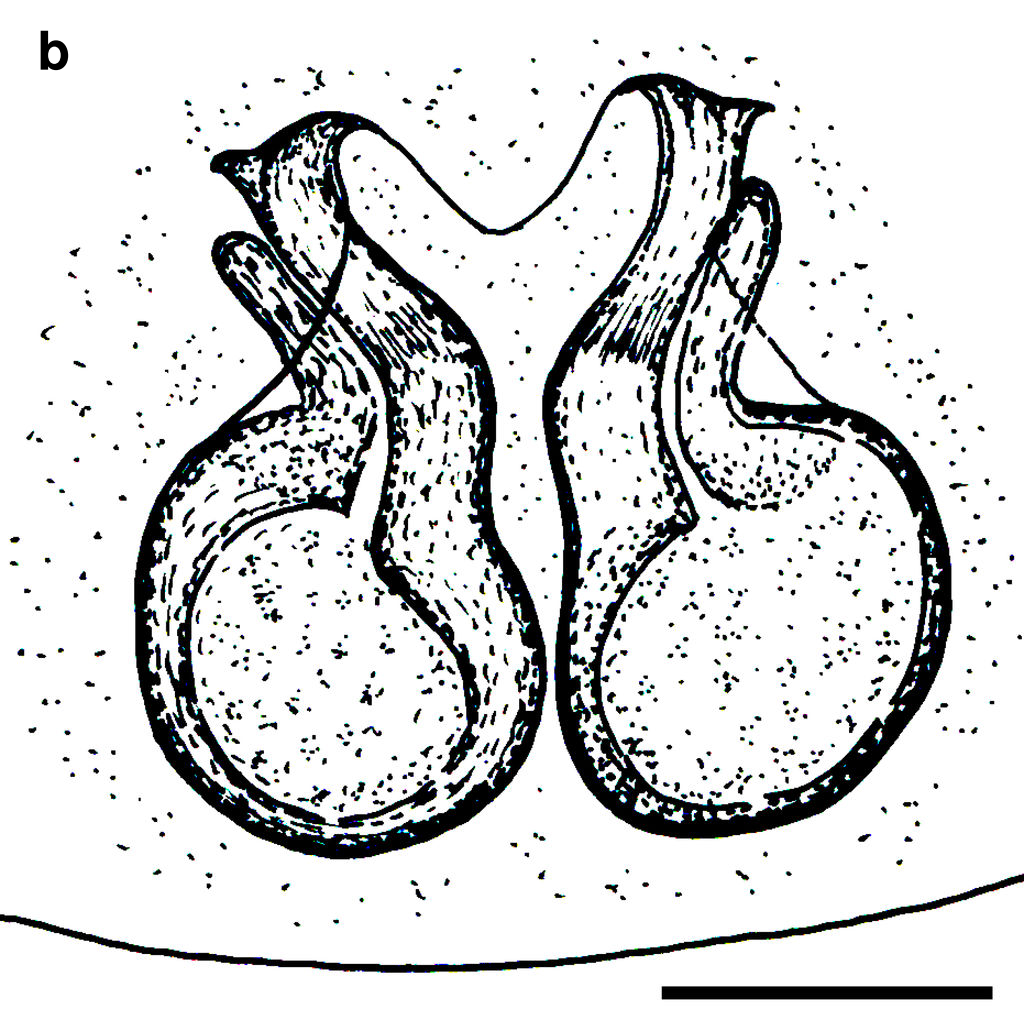
*Heser aradensis*, ventral view.

**Figure 6c.**
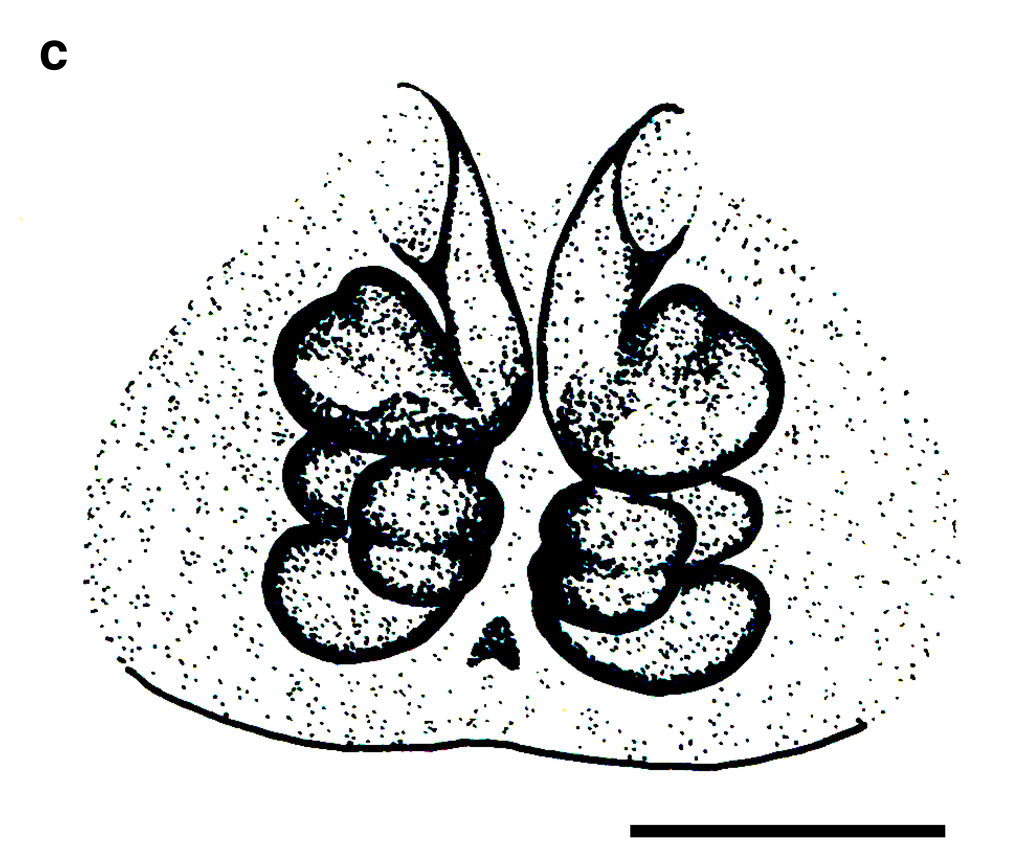
*Heser stoevi* sp. n., paratype, dorsal view.

**Figure 6d.**
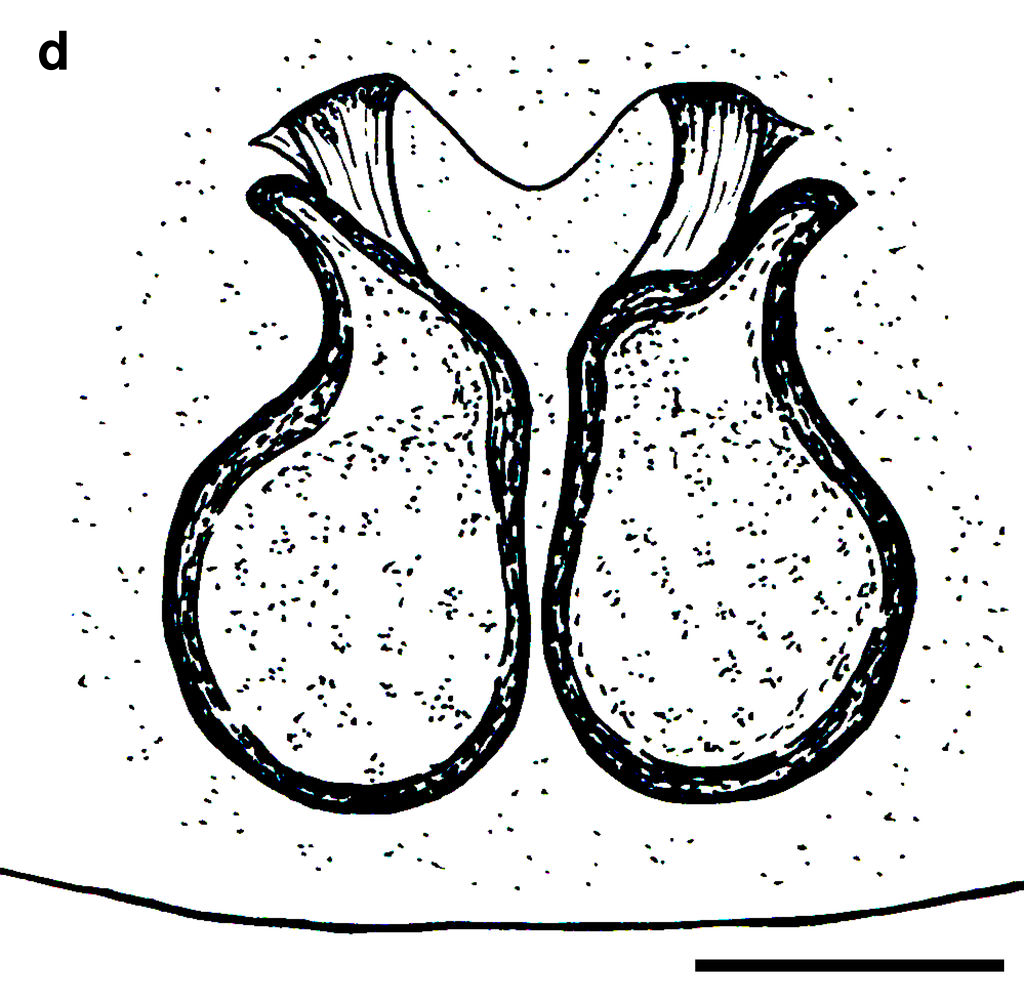
*Heser aradensis*, dorsal view.

**Table 1.** *Heser stoevi* sp. nov., leg measurements (holotype).

**Legs**	**Femur**	**Patella**	**Tibia**	**Metatarsus**	**Tarsus**	**Total**
I	-	-	-	-	-	-
II	1.58	0.98	1.20	1.05	0.83	5.64
III	1.35	0.75	0.90	1.13	0.75	4.88
IV	1.95	1.05	1.50	1.73	0.98	7.21

**Table 2.** *Heser stoevi* sp. nov., leg measurements (paratype).

**Legs**	**Femur**	**Patella**	**Tibia**	**Metatarsus**	**Tarsus**	**Total**
I	1.65	1.05	1.28	0.9	0.75	5.63
II	1.05	0.75	1.05	0.9	0.68	4.43
III	1.13	0.75	0.75	0.9	0.68	4.21
IV	1.8	0.9	1.43	1.35	0.75	6.23
